# The Trend of Changes in Adiponectin, Resistin, and Adiponectin–Resistin Index Values in Type 2 Diabetic Patients with the Development of Metabolic Syndrome

**DOI:** 10.3390/medicina60111795

**Published:** 2024-11-01

**Authors:** Almir Fajkić, Rijad Jahić, Malik Ejubović, Miralem Đešević, Amira Jagodić Ejubović, Orhan Lepara

**Affiliations:** 1 Department of Pathophysiology, Faculty of Medicine, University of Sarajevo, 71000 Sarajevo, Bosnia and Herzegovina; 2 University Clinical Center Sarajevo, 71000 Sarajevo, Bosnia and Herzegovina; rijadjahic2005@gmail.com; 3 Department of Internal Medicine, Cantonal Hospital Zenica, 72000 Zenica, Bosnia and Herzegovina; ejubovic.malik@gmail.com (M.E.); amira_jagodic@hotmail.com (A.J.E.); 4 Department of Cardiology, Polyclinic Eurofarm Center Sarajevo, 71000 Sarajevo, Bosnia and Herzegovina; miralem.desevic@gmail.com; 5 Department of Human Physiology, Faculty of Medicine, University of Sarajevo, 71000 Sarajevo, Bosnia and Herzegovina; orhan.lepara@mf.unsa.ba

**Keywords:** type 2 diabetes mellitus, metabolic syndrome, adiponectin, resistin, AR index

## Abstract

*Background and Objectives*: This study aimed to investigate the novel adiponectin–resistin (AR) index as a predictor of the development of metabolic syndrome (MetS) in individuals with type 2 diabetes mellitus (T2DM). MetS is common in T2DM and increases cardiovascular risk. Adiponectin and resistin, adipokines with opposing effects on insulin sensitivity and inflammation, make the AR index a potential marker for metabolic risk. *Materials and Methods*: This prospective observational study included 80 T2DM participants (ages 30–60) from Sarajevo, Bosnia and Herzegovina, over 24 months. The participants were divided into two groups: T2DM with MetS (*n* = 48) and T2DM without MetS (*n* = 32). Anthropometric data, biochemical analyses, and serum levels of adiponectin and resistin were measured at baseline and every six months. The AR index was calculated using the formula AR = 1 + log10(R) − 1 + log10(A), where R and A represent resistin and adiponectin concentrations. Logistic regression identified predictors of MetS. *Results*: T2DM patients who developed MetS showed a significant decline in adiponectin levels (40.19 to 32.49 ng/mL, *p* = 0.02) and a rise in resistin levels (284.50 to 315.21 pg/mL, *p* = 0.001). The AR index increased from 2.85 to 2.98 (*p* = 0.001). The AR index and resistin were independent predictors of MetS after 18 months, with the AR index showing a stronger predictive value (*p* = 0.007; EXP(B) = 1.265). *Conclusions*: The AR index is a practical marker for predicting MetS development in T2DM participants, improving metabolic risk stratification. Incorporating it into clinical assessments may enhance early detection and treatment strategies.

## 1. Introduction

Type 2 diabetes mellitus (T2DM) is a prevalent chronic condition characterized by insulin resistance and elevated blood sugar levels. This disease poses a significant global health challenge, as it is intricately linked to a variety of complications, particularly cardiovascular diseases. Metabolic syndrome (MetS) is a cluster of conditions, including abdominal obesity, dyslipidemia, hypertension, and insulin resistance. These conditions often coexist with T2DM, significantly increasing the risk of cardiovascular morbidity and mortality. MetS affects between 70% and 80% of individuals with T2DM worldwide, leading to a predisposition to the development of complications of T2DM, such as diabetic nephropathy, diabetic neuropathy, or diabetic retinopathy as the most common [1,2,3].

Components of MetS, particularly abdominal obesity, are associated with chronic low-grade inflammation. This inflammation further worsens insulin resistance and contributes to the development of atherosclerosis. The inflammatory processes underlying MetS can thus create a vicious cycle that deteriorates metabolic control and cardiovascular health in T2DM patients [4].

Adipose tissue plays a crucial and multifaceted role in developing MetS in patients with T2DM. Often considered merely a storage site for excess energy, adipose tissue is, in fact, a dynamic endocrine organ that secretes a variety of hormones and cytokines, collectively known as adipokines. These adipokines, including adiponectin and resistin, significantly influence metabolic processes and contribute to the pathogenesis of MetS in T2DM patients [5]. Adipose tissue, particularly visceral fat, is a significant source of pro-inflammatory cytokines such as tumor necrosis factor-alpha (TNF-α) and interleukin-6 (IL-6). These cytokines induce chronic low-grade inflammation, a hallmark of T2DM and MetS. This persistent inflammation further impairs insulin signaling, worsening insulin resistance and promoting hyperglycemia and other metabolic abnormalities [6].

Although there are intriguing methods for the creation of a diagnostic algorithm of insulin resistance as a key hallmark in the pathophysiology of MetS, it is interpreted as a true challenge for final diagnosis in clinical practice. Previously researched laboratory indices are widely available but their potential for positive discrimination on the purpose of MetS diagnosis is extremely low and variable, simultaneously presenting a lack of a clear cutoff value with a diagnostic potential, besides the already researched AR index [7].

Adiponectin, one of the key adipokines secreted by adipose tissue, is known for its anti-inflammatory and insulin-sensitizing properties. In individuals with T2DM and MetS, adiponectin levels are often markedly reduced. This reduction exacerbates insulin resistance and promotes systemic inflammation, both of which are central features of MetS. Low adiponectin levels have been linked to increased cardiovascular risk and the progression of other MetS components [8]. Conversely, resistin, another critical adipokine, promotes insulin resistance and inflammation. Elevated resistin levels are commonly observed in obese individuals and those with T2DM, contributing to the metabolic disturbances characteristic of MetS [9].

The opposing actions of adiponectin and resistin highlight the complex regulatory role of adipose tissue in metabolic health. The novel adiponectin–resistin (AR) index integrates the inflammatory and insulin-sensitizing properties of adiponectin and resistin, providing a holistic marker of inflammation and insulin resistance due to the importance of the aforementioned dual perspective in conditions like T2DM and MetS appreciating a positive correlation of AR index with the risk of developing T2DM and MetS [10].

However, the aforementioned research papers did not consider potential interactions of the AR index in the development of MetS for patients who already present T2DM and did not provide a study design that prospectively included adiponectin and resistin independently in several time cycles, enhancing the AR index’s ability to reflect the balance of adipokine-mediated effects on insulin sensitivity in terms of MetS and T2DM pathogenesis.

The main objective of this study was to investigate the role of the novel AR index as a predictor of the development of metabolic syndrome in individuals with type 2 diabetes mellitus.

## 2. Materials and Methods

### 2.1. Study Population and Design

This prospective observational clinical study was conducted over 24 months, from May 2016 until May 2018. The study protocol was presented and approved by the Medical Ethical Committees of the Faculty of Medicine, University of Sarajevo in Bosnia and Herzegovina (decision number: 02-03-4-4943/13 from 6 November 2013). Informed consent was obtained from all participants before the commencement of this study. The primary selection of participants was performed based on the criteria for diagnosing DM without MetS. Initially, we recruited 162 consecutive participants (87 men and 75 women) with confirmed T2DM from the Diabetes Counseling Ambulances of the Public Institution Health Center and Endocrinology Clinics in Sarajevo Canton. T2DM participants were recruited from outpatient diabetes counseling clinics. These participants received regular follow-up care for their condition and were identified through their medical history and clinical records maintained at the clinics. All participants were ambulatory patients, and recruitment was based on their clinical diagnosis of T2DM and their eligibility according to this study’s inclusion criteria.

**Figure 1:**
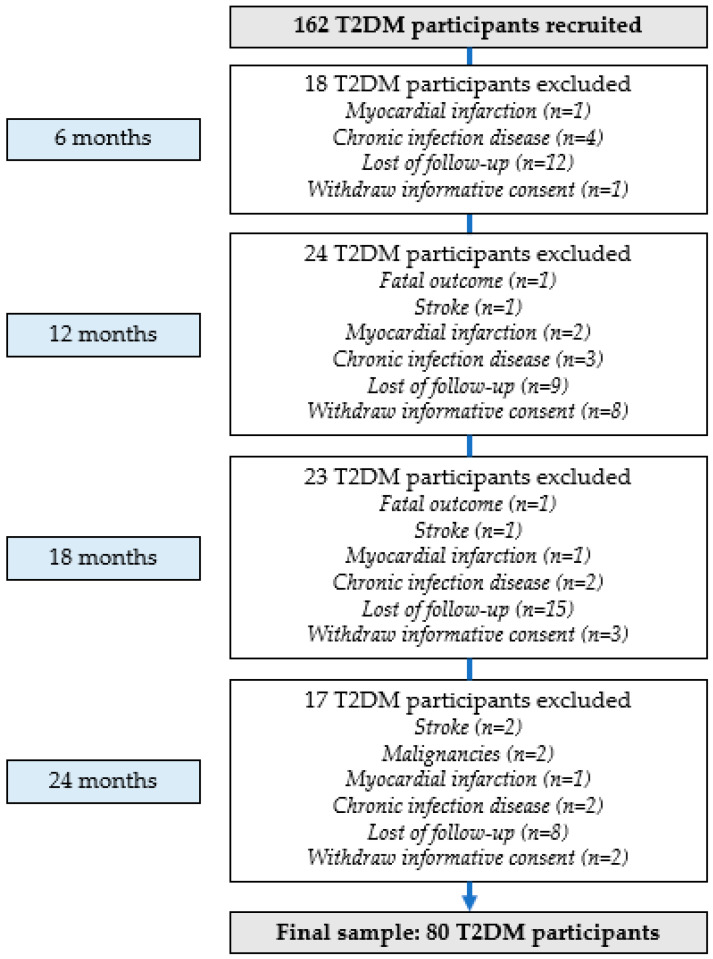
T2DM participants’ selection flowchart.

Eighty-two participants were excluded during this study’s implementation period. The reasons for the exclusion of respondents from this study were as follows: stroke (*n* = 4), myocardial infarction (*n* = 5), newly discovered malignancies (*n* = 2), chronic infectious diseases (*n* = 11), fatal outcome (*n* = 2), lost to follow-up (*n* = 44), and withdrawn informative consent (*n* = 14). After 24 months, 80 participants (41 men and 39 women) met the study criteria, and their data were analyzed (Figure 1).

At the end of the research period, T2DM participants were divided into two groups:

1. T2DM without MetS—participants who did not develop MetS (*n* = 32);
2. T2D with MetS—participants who developed MetS (*n* = 48).

MetS distribution among T2DM participants was as follows:

- 0 months: 0 participants;
- 6 months: 14 participants (17.5%);
- 12 months: 26 participants (32.5%); cumulative 40 (50%);
- 18 months: 8 participants (10%); cumulative 48 (60%);
- 24 months: no new cases.

MetS was diagnosed based on at least three of the five criteria from the National Cholesterol Education Program (NCEP) Adult Treatment Panel III (ATP III) [11]:

- Central obesity: waist circumference (WC): men >102 cm, women >88 cm;
- Triglycerides ≥1.7 mmol/L;
- HDL cholesterol: men ≤ 1.03 mmol/L, women ≤ 1.29 mmol/L;
- Blood pressure: ≥139/≥89 mm Hg;
- Fasting glucose: ≥6.1 mmol/L.

This study included participants aged 30 to 60 years who had a clinical diagnosis of T2DM but had not yet developed MetS at the time of enrollment. To be included, participants had to have a Body Mass Index (BMI) between 18.5 and 24.9 and receive oral antidiabetic therapy. The exclusion criteria were as follows: participants who developed cardiovascular diseases, malignant conditions, or infectious diseases during this study were excluded, as well as those taking medications known to affect serum adiponectin and resistin levels, such as corticosteroids and hormonal contraceptives. Participants with incomplete data were also excluded.

In a two-year follow-up period, detailed data from all T2DM participants, including demographics, medical history, anthropometric measurements, and biochemical analyses, were collected at baseline and every six months.

### 2.2. Anthropometric and Other Measurements

Body weight was measured using a digital scale with 0.1 kg accuracy in the morning before food or drink intake. Height was measured with a stadiometer, with participants barefoot and standing straight. BMI was calculated by dividing weight (kg) by height squared (m^2^). Waist circumference was measured at the umbilicus, and hip circumference at the widest part of the hips; both were recorded in centimeters (cm). Blood pressure was measured with a mercury sphygmomanometer on the dominant arm after a 5 min rest, avoiding coffee or alcohol for 30 min prior. The results were recorded in mmHg.

### 2.3. Biochemical Analyses

Blood samples were collected after an overnight fast and a thorough physical examination. Following coagulation and centrifugation, serum was extracted and stored at −80 °C for subsequent biochemical, adiponectin, and resistin assays. Biochemical parameters were measured using standard methodologies.

Fasting glucose concentration was determined using the glucose oxidase method, while HbA1c levels were assessed via high-performance liquid chromatography.

Lipid profiles were analyzed using an automated system with established protocols: total cholesterol was quantified by the cholesterol oxidase assay, HDL cholesterol (HDL-C) was measured by a direct enzymatic method, and triglycerides were assessed through enzymatic hydrolysis and glycerol measurement. Low-density lipoprotein cholesterol (LDL-C) was calculated using the Friedewald equation, and very-low-density lipoprotein cholesterol (VLDL-C) was derived from triglyceride levels using the formula VLDL = triglycerides/5 [12].

Serum C-reactive protein (CRP) levels were quantified using an immunoturbidimetric assay (Beckman Synchron LX System).

### 2.4. Serum Adiponectin and Resistin Analysis

Serum adiponectin and resistin were measured using ELISA, with the Elabscience Adiponectin ELISA Kits, which have a detection range of 3.906–250 ng/mL and a detection limit of 2.344 ng/mL, and the Elabscience Resistin ELISA Kit, which has a detection range of 31.25–2000 pg/mL and a detection limit of 18.75 pg/mL [13].

### 2.5. AR Index Calculation

The AR index was calculated using the following formula [14]:

AR = 1 + log_10_ (R) − 1 + log_10_ (A)

A is the adiponectin concentration (ng/mL), and R is the resistin concentration (pg/mL).

### 2.6. Statistical Analysis

The statistical analysis of the obtained data was performed using IBM SPSS software (Version 21) [15]. The Kolmogorov–Smirnov test (for samples larger than 50 participants) and the Shapiro–Wilk test (for samples smaller than 50 participants) were used to determine the distribution of continuous variables. Non-parametric tests (Wilcoxon Signed Ranks Test, Friedman Test) were used to test the significance of differences in repeated measurements of continuous variables. The non-parametric Mann–Whitney test was used to test the significance of differences in continuous variables between groups. Spearman’s correlation test determined the relationships’ existence, direction, and strength among continuous variables. Binary logistic regression models were used to examine independent predictors for MetS development at different time intervals (6, 12, 18, and 24 months). The accepted level of statistical significance was *p* < 0.05.

## 3. Results

### Participants Characteristics

Eighty participants (41 males (51.25%) and 39 females (48.75%)) with T2DM were included in this study. Table 1 shows the clinical and biochemical characteristics at the beginning of this study.

A comparative analysis of the baseline values of participants stratified according to the presence of MetS after 24 months revealed significant differences in BMI (*p* = 0.016), fasting glucose levels (*p* = 0.007), HbA1c (*p* = 0.001), CRP (*p* < 0.001), triglycerides (*p* = 0.007), LDL-C (*p* = 0.022), VLDL-C (*p* = 0.007), adiponectin (*p* < 0.001), resistin (*p* < 0.001), and the AR index (*p* < 0.001) (Table 2).

The serum concentration of adiponectin in T2DM participants who developed MetS at the end of this study was 32.49 (31.97–34.03) ng/mL, significantly lower (*p* = 0.02) compared to the serum concentration of adiponectin before the onset of MetS [40.19 (39.27–41.32) ng/mL]. The serum concentration of resistin and AR index values in T2DM participants who developed MetS [315.21 (310.29–319.62) pg/mL; 2.98 (2.97–3.00)] at the end of this study were significantly higher (*p* = 0.001) compared to the values before the onset of MetS [284.50 (278.93–289.12) pg/mL; 2.85 (2.83–2.86)] (Table 3).

In the comparative analysis conducted over the follow-up period (6 months, 12 months, 18 months, and 24 months), the values of adipokines (adiponectin, resistin, and the AR index) significantly differed between T2DM participants with and without MetS at all measurement points (Table S1).

**Figure 2:**
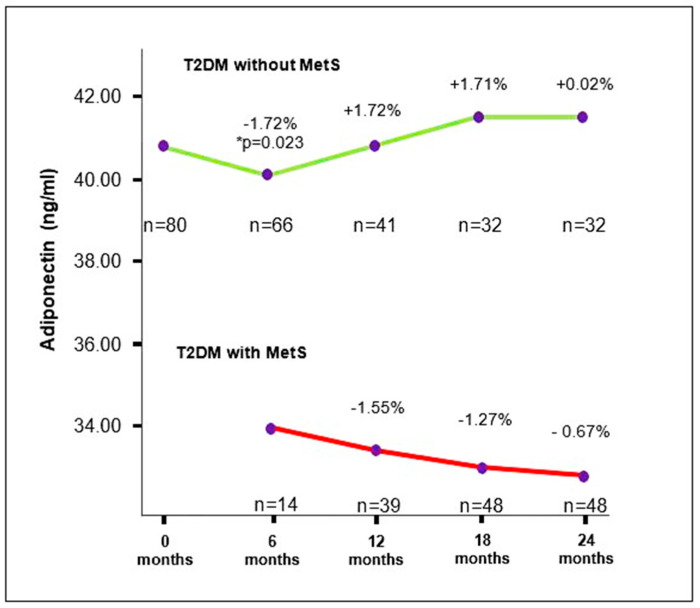
Trends of changes in serum adiponectin concentrations in T2DM participants with and without MetS. The results are presented in percentages (%); T2DM: type 2 diabetes mellitus; MetS: metabolic syndrome; *n*: number of participants. * *p* < 0.05.

The adiponectin concentration in T2DM participants without MetS at the first measurement was 41.4 ± 1.31 ng/mL, significantly higher than that of adiponectin measured secondly (40.07 (38.5–35.2) ng/mL; *p* = 0.023). There was no significant difference in the concentration of adiponectin measured at the third measurement compared to the fourth and fifth measurements, nor between the concentrations of adiponectin measured at the fourth measurement and those at the fifth measurement. In participants with T2DM who developed MetS during this study, a downward trend in serum adiponectin concentration was observed over the 24-month follow-up period, but without a significant difference in adiponectin concentrations between individual observation periods (Figure 2, Table 2 and Table S1).

**Figure 3:**
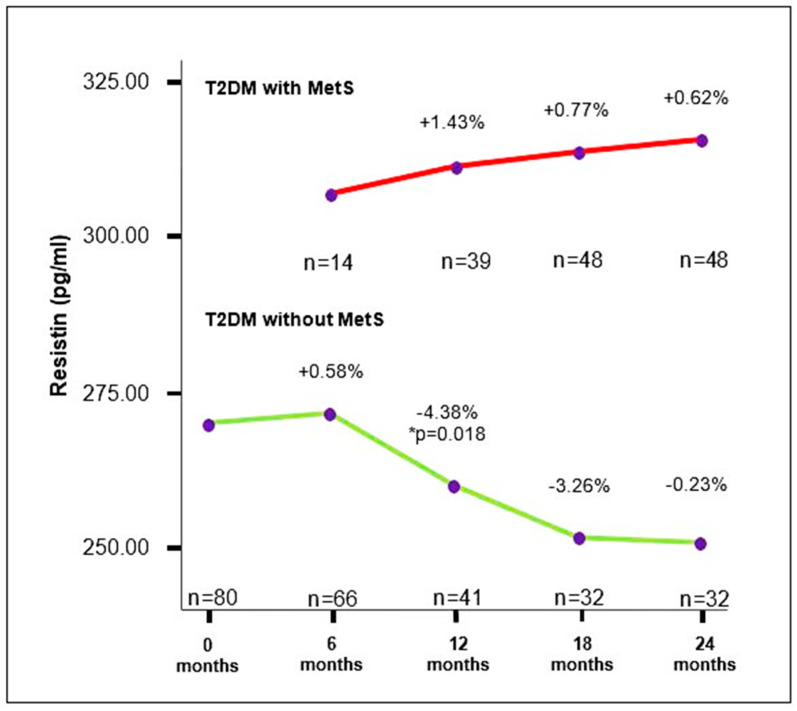
Trends of changes in serum resistin concentrations in T2DM participants with and without MetS. The results are presented in percentages (%); T2DM: type 2 diabetes mellitus; MetS: metabolic syndrome; *n*: number of participants; * *p* < 0.05.

The serum concentration of resistin in T2DM participants without MetS at the first measurement was 269.1 ± 15.8 pg/mL, showing a slight increase at the second measurement, followed by a decreasing trend in subsequent observation periods. A significant decrease in resistin concentration was observed at the third measurement ((259.9 (244.9–272.1) pg/mL) compared to the second measurement (274.6 (354.4–292.4)) pg/mL; *p* = 0.018). In T2DM participants with MetS during this study, a trend of increasing serum resistin concentration over the 24-month follow-up period was noted. However, there was no significant difference in resistin concentrations between individual observation periods (Figure 3, Table 2 and Table S1).

**Figure 4:**
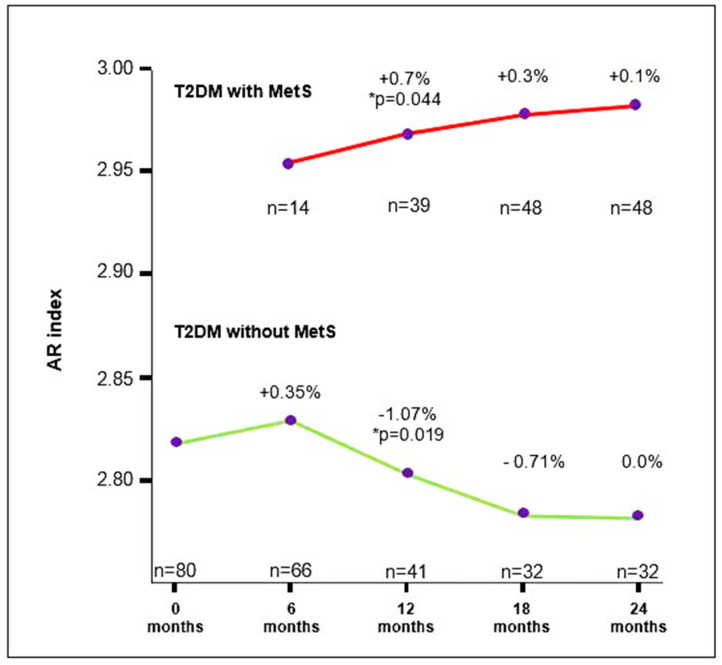
Trends of changes in AR index values in T2DM participants with and without MetS. The results are presented in percentages (%); T2DM: type 2 diabetes mellitus; MetS: metabolic syndrome; *n*: number of participants; * *p* < 0.05.

The AR index in T2DM participants without MetS at the first measurement was 2.78 (2.75–2.80), lower than the value determined at the second measurement (2.83 (2.7–2.8)). After the second measurement, a decrease in the AR index value was observed, with a significantly lower value at the third measurement (2.71 (2.7–2.8)) compared to the second measurement (*p* = 0.019). In T2DM participants who developed MetS during this study, a trend of increasing AR index values was noted over the 24-month follow-up period. A significantly higher AR index value was observed at the third measurement (2.97 (2.95–2.98)) compared to the AR index value at the second measurement (2.95 (2.93–2.97); *p* = 0.044) (Figure 4, Table 2 and Table S1).

To investigate independent predictors of MetS in T2DM participants, the following variables were included in a logistic regression analysis model: adiponectin, resistin, AR index, gender (female), age, T2DM duration, smoking status, CRP, glucose, triglycerides, HDL-c, SBP, and DBP.

In the logistic regression analysis model, examining predictors of MetS after 6 months of observation, it was found that none of the analyzed factors significantly influenced the occurrence of MetS (*p* > 0.05). The model was statistically significant (χ^2^(8) = 93.05, *p* = 0.0001) and could explain 92.9% of the variance in results (Nagelkerke R^2^) and correctly classify 60.0% of cases (Table S2).

After 12 months of observation, it was found that none of the analyzed factors significantly influenced the occurrence of MetS (*p* > 0.05). The model was statistically significant (χ^2^(8) = 24.62; *p* = 0.003) and could explain 53.2% of the variance in results (Nagelkerke R2) and correctly classify 91.3% of cases (Table S3).

Analyzing predictors of MetS after 18 months of observation, it was found that the AR index (*p* = 0.007; EXP(B) = 1.265; 1.067–1.500) and resistin (*p* = 0.015; EXP(B) = 1.301; 1.053–1.606) were independent positive predictors of MetS. The model was statistically significant (χ^2^(8) = 72.58; *p* = 0.0001) and could explain 79.5% of the variance in results (Nagelkerke R2) and correctly classify 92.5% of cases (Table 4).

The number of cases after 24 months of observation was the same as after 18 months, and the logistic regression model showed the same results as in Table 4.

After stratifying the participants based on gender in the logistic regression analysis model and examining predictors of MetS after 6, 12, 18, and 24 months, it was found that none of the analyzed factors significantly influenced the occurrence of MetS (*p* > 0.05).

## 4. Discussion

Our study’s findings on changes in resistin and AR index values in T2DM participants toward the development of MetS have significant implications for healthcare strategies in the face of this global health crisis. This is the first prospective study to provide data about changes in adiponectin and resistin together with their AR index over time for T2DM participants who developed MetS during the observational period. No previous research papers have considered the same topic as described in the results.

Adipokines play a vital role in developing MetS and T2DM by promoting insulin resistance. Key adipokines such as leptin, resistin, and ghrelin disrupt insulin signaling, inhibit glucose receptor activity, and contribute to hyperglycemia. This increase in blood sugar triggers inflammation through reactive oxygen species. Hyperinsulinemia caused by insulin resistance also impairs phagocytic cells, allowing bacterial antigens to activate leukocytes and adipocytes. Subsequently, these cells release proinflammatory cytokines, further aggravating inflammation [16]. Through pro-inflammatory and anti-inflammatory mechanisms, adipokines influence metabolic disorders, which have complex and sometimes contradictory effects. For example, resistin and adiponectin have differing impacts on inflammation and insulin resistance, but both are crucial in the progression of disorders like MetS and T2DM. However, due to the opposite effects on the pathophysiology of T2DM and MetS by adiponectin and resistin, their real applicability would be fulfilled only if practitioners could simultaneously combine both values in laboratory analysis, such as the AR index [10].

Adiponectin is decreased concomitantly with a higher risk of T2DM, as was suggested by Li et al. in meta-analysis studies with a total of 14 598 participants from 13 studies across different including diversified geographical and biological backgrounds in a dose–response relationship [17]. The Whitehall II study by Tabak et al. found that adiponectin levels might fluctuate a decade before T2DM diagnosis and clinical sign presentation [18].

Contrary to previous research paradigms about adiponectin, our results did not show a statistically significant role in predicting the development of MetS in T2DM participants, which could be attributed to the fact that the level of serum adiponectin concentration might be influenced by other metabolic factors (e.g., adipocytes’ turnover rate). Hoffstedt et al. did not find a statistically significant difference in the production of adiponectin levels and serum adiponectin levels for patients with IR, explaining that there was interindividual variation in the range between 8% and 16% for serum adiponectin and insulin sensitivity [19]. Abdella et al. conducted clinical research on adiponectin levels in T2DM patients with IR and without IR. Their results suggested that the adiponectin level in individuals with uncontrolled glycemic status was decreased compared to individuals whose glycemic status was controlled. Additionally, individuals with T2DM and IR had significantly lower levels of adiponectin compared to T2DM individuals without IR [20].

Moreover, hyperglycemia causes dysregulation in the axis of fibroblast growth factor 21 (FGF21) and adiponectin. It principally acts on a decrease in adiponectin, while FGF21 loses its capacity to regulate adiponectin production. Additionally, FGF21 is further increased by hyperglycemia, showing a positive reaction to PPAR gamma. The ligands of PPAR gamma are known for their role in weight loss [21,22].

Considering the FGF family, whose signaling molecules are produced by monocytes and driven by the inflammatory response, an additional potential link between adipokines and MetS might be found in fibroblast growth factor 23 (FGF23). FGF23 increases when the adiponectin level is lowered due to a changed physiological status. Contrary to the adiponectin–FGF23 relation, an increase in the FGF23 level directly produces an increased resistin production, leading to a directly proportional relationship [23,24]. T2DM, per se, leads to a substantial predisposition to developing comorbidities, especially those associated with blood vessels. Recent research indicates that FGF23 directly impacts endothelial dysfunction, leading to a predisposition to endovascular diseases, recognized as key contributors to atherosclerosis, a significant hallmark of T2DM [25].

Previous research has not clearly explained the occurrence of hyper-resistinemia in metabolic disorders like T2DM and MetS. One view links it to resistin gene polymorphism, while another points to inflammation’s role in increasing resistin secretion. The endotoxin lipopolysaccharide (LPS) significantly boosts resistin production through inflammatory cytokines such as TNF-α. This rise can be blocked by aspirin and rosiglitazone, which have anti-inflammatory and insulin-sensitizing effects by inhibiting NF-kB. Inhibiting NF-kB counteracts LPS-induced resistin production [26].

Regarding the role of resistin in T2DM, it was concluded that results showed that resistin levels were increased in T2DM participants compared to the control healthy group and were not directly related to the increased level of BMI [27]. However, considering the role of resistin as a milestone of MetS, its production shows that serum resistin levels strongly correlate with the expression of resistin mRNA in adipose tissue. This expression is increased in MetS, in contrast to adiponectin, whose mRNA is decreased in adipose tissue. Nevertheless, the same research also suggested that resistin might not be a key link between MetS and obesity, as resistin levels were not statistically different between groups of patients with T2DM and MetS. Given the previously mentioned data on the roles of resistin and adiponectin, it is foreseeable that these two parameters must be paired and considered when analyzing their role in T2DM patients with MetS. The AR index represents an accessible toolkit for integrating these parameters [18,28].

Resistin levels have a strong genetic influence on MetS risk, as previous research suggests that serum resistin levels strongly correlate with genetic predisposition via different genotypes in the resistin gene (REST). Research for a correlation between serum adiponectin levels and genetic predisposition still lacks a predictive role for serum adiponectin levels of certain genotypes of the adiponectin gene for MetS [29,30].

The opposing effects of adiponectin and resistin suggest that their interaction may shape the metabolic profile of obese individuals and contribute to obesity-related complications. The AR index is proposed as a metabolic risk indicator in obesity and is positively correlated with the risk of developing T2DM and MetS [10]. In the case–control study, which included 809 participants where, besides control groups, participants had been classified according to the presence of MetS, T2DM, or both diseases, the AR index appeared to statistically significantly correlate with markers of MetS and T2DM and MetS rather than adiponectin and resistin levels alone [14].

Despite the still rare use of adipokines in clinical practice for diagnosis, acute coronary syndrome (ACS) is related to the most common causes of cardiac arrest and lethal outcomes in the world. The AR index was proven to be the best independent predictor of ACS between analyzed inflammatory cytokines and adipokines considered per se as a predictor (actual adipokines alone did not have statistical significance in ACS diagnosis) [31].

In terms of potential intervention due to altered levels of adiponectin and resistin in T2DM concerning metabolic factors, atorvastatin, as the example of statin therapeutic intervention with antioxidant potential, did not influence the status of adiponectin and resistin, as suggested in research by Shetty et al. Additionally, in the aforementioned cross-sectional study, they noticed that despite decreased CRP levels with atorvastatin intervention, which might implicate that inflammation is not a mechanism by which adipokines interact in human pathophysiology [32].

As described earlier, this research focused on a population aged between 30 and 60 years because the production of adipokines is related to human aging. It is known that stem cells and progenitor cells from adipose tissue increase during the aging period between 30 and 50 years. Defects in these cells may represent a key factor in developing insulin resistance. As individuals reach ages over 50, continuing up to 60 years, there is a natural decline in adipokine production, which may further influence insulin resistance as a physiological process [33]. However, low BMI status in a population older than 60 might influence adiponectin levels, displaying a falsely increased level and further promoting incorrect pathological status [34]. Therefore, regarding MetS criteria, this study included only participants between 30 and 60 years of age due to the higher prevalence of MetS development reaching a peak between 50 and 60 [35].

Regarding biostatistics methods, the AR index has an advantage over the AR or RA ratio because it applies logarithmic values. This representation offers a more precise value than a simple ratio, as different activities of individuals with fatty tissue can lead to incorrect deductions when using a simple ratio. Therefore, with the AR index formula mentioned above, the terminology states that the AR index is not crucial, as the results remain the same despite the interchange of the first and second positions.

Due to research in geographical areas where the white race is dominant, it would be interesting to provide data about adipokines (adiponectin and resistin encountering AR ratio) in other race variants for further research.

Our study has several key strengths that significantly contribute to MetS and T2DM research. First and foremost, introducing the AR index as a novel biomarker for predicting the development of MetS in patients with T2DM represents an innovative approach. By combining the effects of two crucial adipokines—adiponectin and resistin—the AR index offers a more comprehensive assessment of metabolic risk, potentially enhancing the accuracy of predictions compared to traditional markers. Furthermore, our study is designed as a prospective study, following participants over 24 months. This design allows for the observation of changes in adipokine levels in real time, providing deeper insights into the dynamics of these biomarkers before the onset of MetS. Such a longitudinal approach strengthens the validity of our findings and their relevance to clinical practice.

Despite its strengths, our study does have some limitations that should be acknowledged. First, limited generalizability is a concern. This study was conducted in a specific geographical region (Sarajevo, Bosnia and Herzegovina) with a relatively homogenous population. As a result, the findings may not be directly applicable to other populations with different ethnic, genetic, or environmental backgrounds. This limits how our results can be generalized to broader, more diverse populations. Second, this study’s small sample size may limit the statistical power and the ability to detect smaller effect sizes, as this prospective observational study may only make associations between variables if it confirms causality. However, the sample was large enough for an initial exploratory analysis, although a larger sample would have given more robust and reliable results, providing greater confidence in the findings and their applicability to a broader population. As such, we did not collect or analyze data on patient treatments and associated comorbidities, as it was beyond the scope of our study. Lastly, as a single-center study, our research is subject to biases related to the specific clinical environment in which it was conducted. Practices and patient characteristics unique to this center may have influenced the results, and multi-center studies are needed to confirm the reproducibility of our findings across different settings and populations.

Therefore, future research should be multi-center and longitudinal, involving more extensive, diverse populations across various ethnicities, to explore the causal link between the AR index and MetS development. Randomized controlled trials (RCTs) could also help evaluate the effects of adipokine-targeting interventions on MetS. Incorporating the AR index as a predictive tool in clinical settings could be insightful, especially when examining its relationship with lifestyle factors like diet and exercise. Future studies should consider the impact of treatments and comorbidities to provide a more comprehensive understanding of the factors contributing to MetS. Advanced statistical approaches, such as mediation or moderation analyses, could shed light on the complex interactions between adiponectin, resistin, and other metabolic elements.

## 5. Conclusions

Integrating the AR index into standard metabolic assessments can enhance early detection, risk stratification, and personalized treatment, ultimately improving patient outcomes. The measurement of the AR index is practical and can be easily integrated into routine clinical assessments. Blood samples required for adiponectin and resistin measurements are commonly collected in patients undergoing metabolic evaluation. Calculating the AR index adds minimal complexity to the diagnostic process but offers significant additional insights.

## Figures and Tables

**Table 1:** General characteristics of the studied population.

Characteristics of Participants (n = 80)	Values
Age (years)	49 (46–52)
T2DM duration (years)	5 (4–7)
Body weight (kg)	76.00 (67.25–81.00)
BMI (kg/m2)	23.91 (23.42–24.89)
WC (cm)(female/male)	80.50 (75.5–86.7)/93.5 (89.0–102.5)
Fasting glucose (mmol/L)	5.70 (5.53–6.00)
HbA1c (%)	5.85 (5.70–6.00)
CRP (mg/L)	2.00 (1.80–2.50)
Cholesterol (mmol/L)	4.60 (4.13–5.08)
Triglycerides (mmol/L)	1.60 (1.20–2.18)
LDL-C (mmol/L)	1.90 (1.60–2.20)
HDL-C (mmol/L)	1.20 (1.00–1.30)
VLDL-C (mmol/L)	0.72 (0.54–0.99)
SBP (mmHg)	130 (120–140)
DBP (mmHg)	80 (80–100)
Adiponectin (ng/dL)	40.97 (39.68–41.51)
Resistin (pg/dL)	276.06 (257.01–285.77)
AR index	2.83 (2.79–2.86)

The results are presented as the median and interquartile range (25–75th percentiles); T2DM: type 2 diabetes mellitus; BMI: body mass index; WC: waist circumference; HbA1C: glycated hemoglobin; CRP: C-reactive protein; LDL-C: low-density lipoprotein cholesterol; HDL-C: high-density lipoprotein cholesterol; VLDL: very-low-density lipoprotein cholesterol; SBP: systolic blood pressure; DBP: diastolic blood pressure; AR: adiponectin–resistin.

**Table 2:** Comparative analysis of baseline participant values stratified by MetS status after 24 months.

Parameters	T2DM Without MetS (*n* = 32)	T2DM with MetS (*n* = 48)	*p*
Age (years)	50.9 ± 6.3	50.6 ± 7.8	0.658
T2DM duration (years)	5.3 (4.6–6.8)	5.5 (4.9–7.0)	0.212
BMI (kg/m^2^)	23.14 ± 1.7	24.5 ± 1.5	0.016
WC (cm) (female/male)	82.7 ± 3.7/92.9 ± 4.0	83.9 ± 4.1/93.6 ± 4.0	0.157/0.117
Fasting glucose (mmol/L)	5.6 (5.5–5.8)	5.8 (5.6–6.1)	0.007
HbA1c (%)	5.8 (5.5–5.9)	6.0 (5.8–6.1)	0.001
CRP (mg/L)	1.9 (1.4–2.1)	2.3 (2.0–2.7)	<0.001
Cholesterol (mmol/L)	4.57 ± 0.53	4.68 ± 0.73	0.472
Triglycerides (mmol/L)	1.2 (1.1–2.0)	1.6 (1.4–2.1)	0.007
LDL-C (mmol/L)	1.8 (1.6–1.9)	1.9 (1.6–2.3)	0.022
HDL-C (mmol/L)	1.2 (1.0–1.3)	1.2 (0.9–1.3)	0.389
VLDL-C (mmol/L)	0.54 (0.5–0.9)	0.7 (0.6–1.0)	0.007
SBP (mmHg)	130.0 (130.0–140.0)	130.0 (130.0–140.0)	0.303
DBP (mmHg)	90.0 (80.0–90.0)	90.0 (80.0–90.0)	0.451
Adiponectin (ng/dL)	41.4 ± 1.31	40.32 ± 1.1	<0.001
Resistin (pg/dL)	269.1 ± 15.8	283.4 ± 10.3	<0.001
AR index	2.78 (2.75–2.80)	2.85 (2.83–2.86)	<0.001

The results are presented as the median and interquartile range (25–75th percentiles); T2DM: type 2 diabetes mellitus; MetS: metabolic syndrome; BMI: body mass index; WC: waist circumference; HbA1C: glycated hemoglobin; CRP: C-reactive protein; LDL-C: low-density lipoprotein cholesterol; HDL-C: high-density lipoprotein cholesterol; VLDL: very-low-density lipoprotein cholesterol; SBP: systolic blood pressure; DBP: diastolic blood pressure; AR: adiponectin–resistin.

**Table 3:** The concentration of adiponectin, resistin, and the AR index value in T2DM participants with MetS at the initial screening and final follow-up.

Parameters	Initial Screening	Final Follow-Up	*p*
Adiponectin (ng/dL)	40.19 (39.27–41.32)	32.49 (31.97–34.03)	0.02
Resistin (pg/mL)	284.50 (278.93–289.12)	315.21 (310.29–319.62)	0.001
AR index	2.85 (2.83–2.86)	2.98 (2.97–3.00)	0.001

The results are presented as the median and interquartile range (25–75th percentiles); AR: adiponectin–resistin.

**Table 4:** Independent predictors of MetS in T2DM participants after 18 months of observation.

Parameter	B	Standard Error	*p*	Exp(B)	95.0% Confidence Interval for Exp(B)
Adiponectin	1.132	0.989	0.252	3.102	0.447–21.529
Resistin	0.263	0.108	0.015	1.301	1.053–1.606
AR index	0.235	0.087	0.007	1.265	1.067–1.500
Gender (female)	−3.470	2.850	0.223	0.031	0.000–8.295
Age	−0.076	0.127	0.551	0.927	0.722–1.189
T2DM duration	−0.164	0.394	0.678	0.849	0.392–1.839
Smoking	−0.190	0.637	0.765	0.827	0.237–2.882
CRP	−0.664	1.041	0.523	0.515	0.067–3.957
Fasting glucose	−0.446	0.769	0.562	0.640	0.142–2.893
WC	0.101	0.097	0.296	1.106	0.915–1.338
Tryglicerides	1.818	2.007	0.365	6.158	0.120–314.842
HDL-C	1.009	2.954	0.733	2.742	0.008–895.481
SBP	0.101	0.541	0.852	1.106	0.383–3.193
DBP	0.852	0.703	0.225	2.345	0.591–9.299
Dependent Variable: Metabolic Syndrome after 18 months of observation					

MetS: metabolic syndrome; AR: adiponectin–resistin; T2DM: type 2 diabetes mellitus; CRP: C-reactive protein; WC: waist circumference; HDL-C: high-density lipoprotein cholesterol; SBP: systolic blood pressure; DBP: diastolic blood pressure.

## Data Availability

Data are available upon reasonable request made to the corresponding author via e-mail.

## Supplementary Materials

The following supporting information can be downloaded at: https://www.mdpi.com/article/10.3390/medicina60111795/s1, Table S1: Comparative analysis of adiponectin. resistin. and AR index values during different follow-up periods (6 months. 12 months. 18 months. and 24 months); Table S2: Independent predictors of MetS in T2DM participants after 6 months of observation; Table S3: Independent predictors of MetS in T2DM participants after 12 months of observation
