# Supplementary material for: The Trend of Changes in Adiponectin, Resistin, and Adiponectin–Resistin Index Values in Type 2 Diabetic Patients with the Development of Metabolic Syndrome
Source: Medicina (Kaunas). 2024 Nov 1;60(11):1795. doi: 10.3390/medicina60111795 (PMC11596469; doi:10.3390/medicina60111795)
Supplement: Supplementary file 1 [file medicina-60-01795-s001.zip › medicina-3249497-supplementary.pdf]

Table S1. Comparative analysis of adiponectin. resistin. and AR index values during different follow-up periods (6 months. 12 months. 18 months. and 24 months).

**Parameters**	**Groups**	**After 6 months**	***p***	**After 12 months**	***p***	**After 18 months**	***p***	**After 24 months**	***p***
Adiponectin (ng/dL)	T2DM without MetS	40.07 (38.5-35..2)	0.007	40.7±1.9	0.007	41.4±1.3	<0.001	41.4±1.3	<0.001
	T2DM with MetS	33.4 (33.0-35.2)		33.4±1.3		33.0±1.5		32.79±1.5	
Resistin (pg/dL)	T2DM without MetS	274.6 (354.4-292.4)	<0.001	259.9 (244.9-272.1)	<0.001	256.0 (242.7-263.6)	<0.001	252.6 (241.2-263.9)	<0.001
	T2DM with MetS	308.0 (298.8-312.5)		310.9 (306.1-316.6)		313.1 (308.2-318.6)		315.1 (310.2-319.5)	
AR index	T2DM without MetS	2.83 (2.7-2.8)	0.006	2.71 (2.7-2.8)	<0.001	2.7±0.04	<0.001	2.78±0.04	<0.001
	T2DM with MetS	2.95 (2.93-2.97)		2.97 (2.95-2.98)		2.9±0.02		2.98±0.02	

The results are presented as the median and interquartile range (25-75th percentiles); T2DM: Type 2 Diabetes Mellitus; MetS: Metabolic Syndrome;AR: adiponectin-resistin

Table S2. Independent predictors of MetS in T2DM participants after 6 months of observation

Parameter	B	Standard error	p	Exp(B)	95.0% Confidence Interval for Exp(B)
Adiponectin	-1.597	0.846	0.157	0.203	0.031-2.089
Resistin	1.533	1.152	0.118	4.627	0.874-24.486
AR index	1.391	0.756	0.073	4.019	0.902-11.432
Gender (female)	-2.411	1.745	0.134	0.089	0.012-1.321
Age	0.108	0.236	0.664	1.115	0.712-2.153
T2DM duration	-0.145	0.895	0.576	0.823	0.313-1.726
Smoking	-0.177	0.856	0.698	0.782	0.142-3.432
CRP	-1.543	0.975	0.078	4.682	0.826-24.356
Fasting glucose	0.076	0.233	0.743	1.079	0.684-1.702
WC	0.161	0.721	0.535	1.175	0.523-2.982
Tryglicerides	0.262	0.789	0.543	1.280	0.284-5.789
HDL-C	-0.753	1.234	0.402	0.582	0.063-5.083
SBP	0.065	0.520	0.595	1.067	0.380-2.740
DBP	0.140	0.570	0.610	1.150	0.410-3.050

Dependent Variable: Metabolic Syndrome after 6 months of observation

MetS: Metabolic syndrome; AR: adiponectin-resistin; T2DM: Type 2 Diabetes Mellitus; CRP: C-reactive protein; WC: Waist Circumference; HDL-C: high-density lipoprotein cholesterol; SBP: systolic blood pressure; DBP: diastolic blood pressure

Table S3. Independent predictors of MetS in T2DM participants after 12 months of observation

Parameter	B	Standard error	p	Exp(B)	95.0% Confidence Interval for Exp(B)
Adiponectin	1.132	0.789	0.196	2.452	0.574-10.475
Resistin	0.763	0.564	0.097	2.145	1.015-4.542
AR index	0.435	0.304	0.078	1.545	1.045-3.876
Gender (female)	-1.470	1.254	0.098	0.473	0.052-4.325
Age	0.076	0.218	0.557	1.067	0.793-2.138
T2DM duration	-0.164	0.314	0.433	0.849	0.422-1.839
Smoking	-0.190	0.637	0.665	0.827	0.237-2.882
CRP	-0.664	1.041	0.493	0.555	0.067-3.957
Fasting glucose	-0.446	0.769	0.565	0.740	0.312-2.893
WC	0.201	0.237	0.482	1.223	0.682-2.325
Tryglicerides	1.118	0.934	0.223	2.842	0.783-10.564
HDL-c	-0.573	0.973	0.245	1.774	0.382-7.243
SBP	0.101	0.541	0.722	1.203	0.543-2.919
DBP	0.652	0.703	0.302	1.745	0.723-5.229
Dependent Variable: Metabolic Syndrome after 12 months of observation					

MetS: Metabolic syndrome; AR: adiponectin-resistin; T2DM: Type 2 Diabetes Mellitus; CRP: C-reactive protein; WC: Waist Circumference; HDL-C: high-density lipoprotein cholesterol; SBP: systolic blood pressure; DBP: diastolic blood pressure
